# Global Sensitivity Analysis of Leaf-Canopy-Atmosphere RTMs: Implications for Biophysical Variables Retrieval from Top-of-Atmosphere Radiance Data

**DOI:** 10.3390/rs11161923

**Published:** 2019-08-17

**Authors:** Jochem Verrelst, Jorge Vicent, Juan Pablo Rivera-Caicedo, Maria Lumbierres, Pablo Morcillo-Pallarés, José Moreno

**Affiliations:** 1 Image Processing Laboratory (IPL), Parc Científic, Universitat de València, 46980 Paterna, Spain; 2 Magellium, 31520 Toulouse, France; 3 CONACyT-UAN, Secretaría de Investigación y Posgrado, Universidad Autónoma de Nayarit, Ciudad de la Cultura Amado Nervo, CP. Tepic 63155, Mexico

**Keywords:** radiative transfer models, global sensitivity analysis, emulation, machine learning, top-of-atmosphere radiance data, PROSAIL, MODTRAN, retrieval, Sentinel-2

## Abstract

Knowledge of key variables driving the top of the atmosphere (TOA) radiance over a vegetated surface is an important step to derive biophysical variables from TOA radiance data, e.g., as observed by an optical satellite. Coupled leaf-canopy-atmosphere Radiative Transfer Models (RTMs) allow linking vegetation variables directly to the at-sensor TOA radiance measured. Global Sensitivity Analysis (GSA) of RTMs enables the computation of the total contribution of each input variable to the output variance. We determined the impacts of the leaf-canopy-atmosphere variables into TOA radiance using the GSA to gain insights into retrievable variables. The leaf and canopy RTM PROSAIL was coupled with the atmospheric RTM MODTRAN5. Because of MODTRAN’s computational burden and GSA’s demand for many simulations, we first developed a surrogate statistical learning model, i.e., an emulator, that allows approximating RTM outputs through a machine learning algorithm with low computation time. A Gaussian process regression (GPR) emulator was used to reproduce lookup tables of TOA radiance as a function of 12 input variables with relative errors of 2.4%. GSA total sensitivity results quantified the driving variables of emulated TOA radiance along the 400–2500 nm spectral range at 15 cm^–1^ (between 0.3–9 nm); overall, the vegetation variables play a more dominant role than atmospheric variables. This suggests the possibility to retrieve biophysical variables directly from at-sensor TOA radiance data. Particularly promising are leaf chlorophyll content, leaf water thickness and leaf area index, as these variables are the most important drivers in governing TOA radiance outside the water absorption regions. A software framework was developed to facilitate the development of retrieval models from at-sensor TOA radiance data. As a proof of concept, maps of these biophysical variables have been generated for both TOA (L1C) and bottom-of-atmosphere (L2A) Sentinel-2 data by means of a hybrid retrieval scheme, i.e., training GPR retrieval algorithms using the RTM simulations. Obtained maps from L1C vs L2A data are consistent, suggesting that vegetation properties can be directly retrieved from TOA radiance data given a cloud-free sky, thus without the need of an atmospheric correction.

## 1 Introduction

Retrieving spatially-explicit vegetation biophysical variables from space is one of the main goals of optical remote sensing, and one of the objectives of international space programs such as NASA Earth Observation Systems or the European Copernicus satellites constellation [1,2]. Particularly, with the Sentinel-2 (S2) constellation an unprecedented inflow of optical data emerged for vegetation monitoring applications with an optimized balance between high spatial, spectral and temporal resolution [3]. The land surface reflectance for retrieval of biophysical variables is estimated from these satellite observations through atmospheric corrections [4,5]. However, accurate atmospheric correction strategies need exact atmospheric variables from the satellite data itself e.g., [6,7] or from external meteorological sources such as AERONET [8] or ECMWF [9]. As the retrieval is based on all kinds of assumptions regarding the model used and the retrieval method applied this step remains challenging, with potentially large uncertainties in the derived atmospheric characteristics and error propagation into surface reflectance [10]. To avoid the limitations of retrieving biophysical variables from surface reflectance data, some studies have demonstrated the possibility to determine biophysical variables directly from at-sensor top-of-atmosphere (TOA) radiance, [11–15] without the necessity to go through the atmospheric correction process [11,12]. The downside of these approaches, however, is that they are not straightforward; they require a sound physical understanding on the factors determining the at-sensor spectral TOA radiance, e.g., as studied in [16–18]. It implies that biophysical variables retrieval from TOA radiance data have so far been restricted to experimental studies. With the purpose of democratizing these approaches to the broader community, what is lacking is a freely available, streamlined and generic processing framework that enables to automate retrieval applications directly from TOA radiance data.

At-sensor spectral TOA radiance is the combination of radiometric effects from surface reflectance, atmospheric effects and target surroundings convolved with the sensor spectral and spatial response functions [19]. Consequently, the identification of the key input variables that drive TOA radiance is a first mandatory step to retrieve biophysical variables directly from at-sensor TOA radiance data. Once having the drivers along the spectral range identified, it opens the door to develop dedicated TOA radiance retrieval algorithms for optical sensors such as S2, taking into account the wavelength-dependent role of the atmospheric factors. These drivers can be theoretically identified by means of coupled surface-atmosphere radiative transfer models (RTMs).

Optical RTMs provide a physical interpretation of light interactions within a medium, e.g., leaf, canopy and atmosphere, and are based on solving the radiative transfer equation. To exploit at-sensor TOA radiance from vegetated surfaces, we need to consider three scales: (1) leaf, (2) canopy and (3) atmosphere; which are associated to two groups of RTMs: vegetation and atmospheric RTMs. Vegetation RTMs study the relationship between leaf and canopy biophysical variables and reflectance, absorbance and scattering mechanisms. The two most widely used models are the leaf model PROSPECT [20], and the canopy model SAIL [21]. The coupling of these two models, named PROSAIL, has been used for over 30 years in sensitivity and retrieval studies [22,23]. Atmosphere RTMs study the interaction of radiation with the atmosphere, on its way to the surface, and reflected back to the sensor. MODTRAN is among the most widely used RTM for atmospheric simulation and correction due to its accurate simulation of the coupled absorption and scattering effects [24,25]. Accordingly, the coupling of PROSAIL with MODTRAN allows assessing the leaf, canopy, and atmosphere variables [11] that drive the observed at-sensor TOA radiance [26,27].

Enabling identifying and quantifying the role of leaf-canopy-atmosphere variables in determining TOA radiance requires a rigorous sensitivity analysis that takes all interactions into account. Such systematic analysis can be achieved by means of a global sensitivity analysis (GSA) [28]. GSA provides information on how the variation of model output is produced by the variation of model input variables individually and globally through interactions with each other [29]. Hence, GSA enables to identify the influential and non-influential input variables for a model output, e.g., TOA radiance along the 400–2500 nm spectral range. The drawback of GSA methods is that they are computationally expensive and complex because of the required large number of model evaluations [28]. This is an important issue when coupled vegetation-atmosphere RTMs are used, since the computational burden of such coupled models can be substantial [19,30]. To overcome this computational burden it has been proposed to make use of emulation [31–33]. Emulators are statistical models that approximate the input-output results of an RTM by means of machine learning [32], and this at a fraction of the RTM computational cost. This technique has been earlier proven successful in GSA studies of advanced physical models in various domains to enable identifying driving variables [19,32,34–37], and will be further explored in this work.

Having entered the era of the Sentinels, the opportunity arises to develop retrieval algorithms directly from S2 L1C data, i.e., at-sensor TOA radiance data. Accordingly, the pursued approach is as follows: first an emulator from a surface-atmosphere model is developed as an approximation of the original RTMs in order to identify the variables through a GSA of TOA radiance in the entire visible and near infrared (VNIR) to shortwave infrared (SWIR) spectral range at a spectral resolution of 1 nm. Based on these GSA results, biophysical variables retrieval strategies applicable directly to an at-sensor TOA radiance dataset will be developed. From past experiences where different retrieval methods have been compared for S2 data at TOC scale [38], the so-called hybrid retrieval methods, i.e., where RTM data is used for training machine learning methods, are particularly promising in terms of accuracy and processing speed [2,39]. Here, the developed retrieval algorithms should eventually be applicable to S2 L1C products, thereby avoiding the uncertainties of the atmospheric correction process [40].

Altogether, this study boils down to the following objectives: (1) to develop emulators to approximate the coupled PROSAIL-MODTRAN RTMs for a set of input variables and TOA radiance output; (2) to apply the emulator into a GSA in order to identify the driving variables; and finally, as a proof of concept, (3) develop hybrid retrieval models for biophysical variables from S2 L1C (TOA radiance) and L2A (bottom-of-atmosphere reflectance) data. All these objectives have been tackled with an in-house developed software framework that is made freely available to the community.

The remainder of this paper is structured as follows. Section 2 gives a further insight into S2 mission with specifications about instrument characteristics and its atmospheric correction (Sen2Cor) and biophysical retrieval algorithms. Section 3 presents the software framework, RTM configurations and toolboxes used to conduct the emulation, GSA and the TOA retrieval performance assessment strategy. This is followed by presenting the results in Section 4 which are discussed in a broader context (Section 5). Section 6 concludes this paper.

## 2 The Sentinel-2 Mission

**Figure 1:**
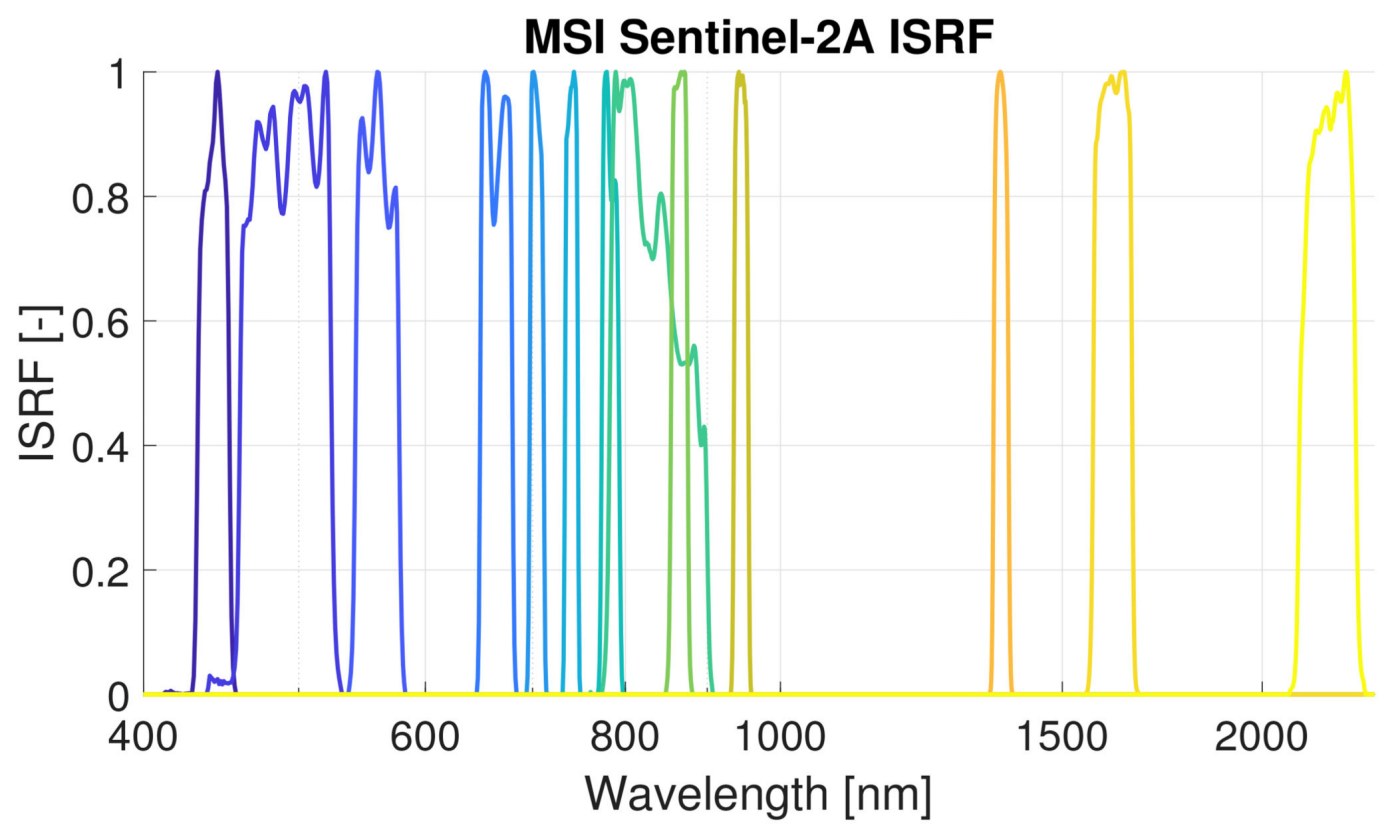
MSI-S2A spectral response function for the 13 spectral bands used by the Ground Segment from 15 January 2018 [42].

Sentinel-2 (S2) is a satellite mission part of the European Commission’s *Copernicus* programme, with the goal of monitoring vegetation, soil and inland and coastal water areas for supporting agro-ecosystems applications [3]. Developed by the European Space Agency (ESA), S2 mission consists of a constellation of two satellites (S2A and S2B) that enables a global revisit time below 5 days. S2’s optical instrument-the MultiSpectral Instrument (MSI)-covers a wide swath (290 km) with high spatial resolution (10–60 m) in 13 spectral bands from the visible and NIR (VNIR) to SWIR spectral range. Further mission technical characteristics are summarized in Table 1 and Figure 1 for band configuration.

The S2 MSI data is freely available from Copernicus Open Access Hub. From mid 2018 onwards, two reflectance products are provided: L1C and L2A. The L1C product refers TOA reflectances (i.e., TOA radiance normalized by incident solar irradiance). The L2A product refers to bottom-of-atmosphere (BOA) reflectance, which is achieved by means of the Sen2Cor atmospheric correction scheme (version 2.4.1) [5]. Sen2Cor processing scheme is based on state-of-the-art algorithms that include cirrus cloud correction and scene classification [43,44]. Sen2Cor relies on the Dark Dense Vegetation algorithm for the retrieval of aerosol type (rural/continental by default) and optical thickness value at 550 nm (AOT_550_) [45]. The Atmospheric Pre-corrected Differential Absorption (APDA) algorithm is implemented for the retrieval of columnar water vapor (CWV) [46]. Derivation of surface reflectance is achieved from the atmospheric inversion of a set of look-up tables generated with the libRadtran atmospheric RTM [47]. Sen2Cor achieves uncertainties around 0.03 for the AOT_550_ and 0.3 gocm^–2^ for the CWV, which are propagated to absolute errors of <0.05 in surface reflectance [48–50]. These errors, nevertheless, should not hamper the retrieval of biophysical variables from L2A reflectance data, e.g., as successfully demonstrated by [51,52].

## 3 Materials and Methods

**Figure 2:**
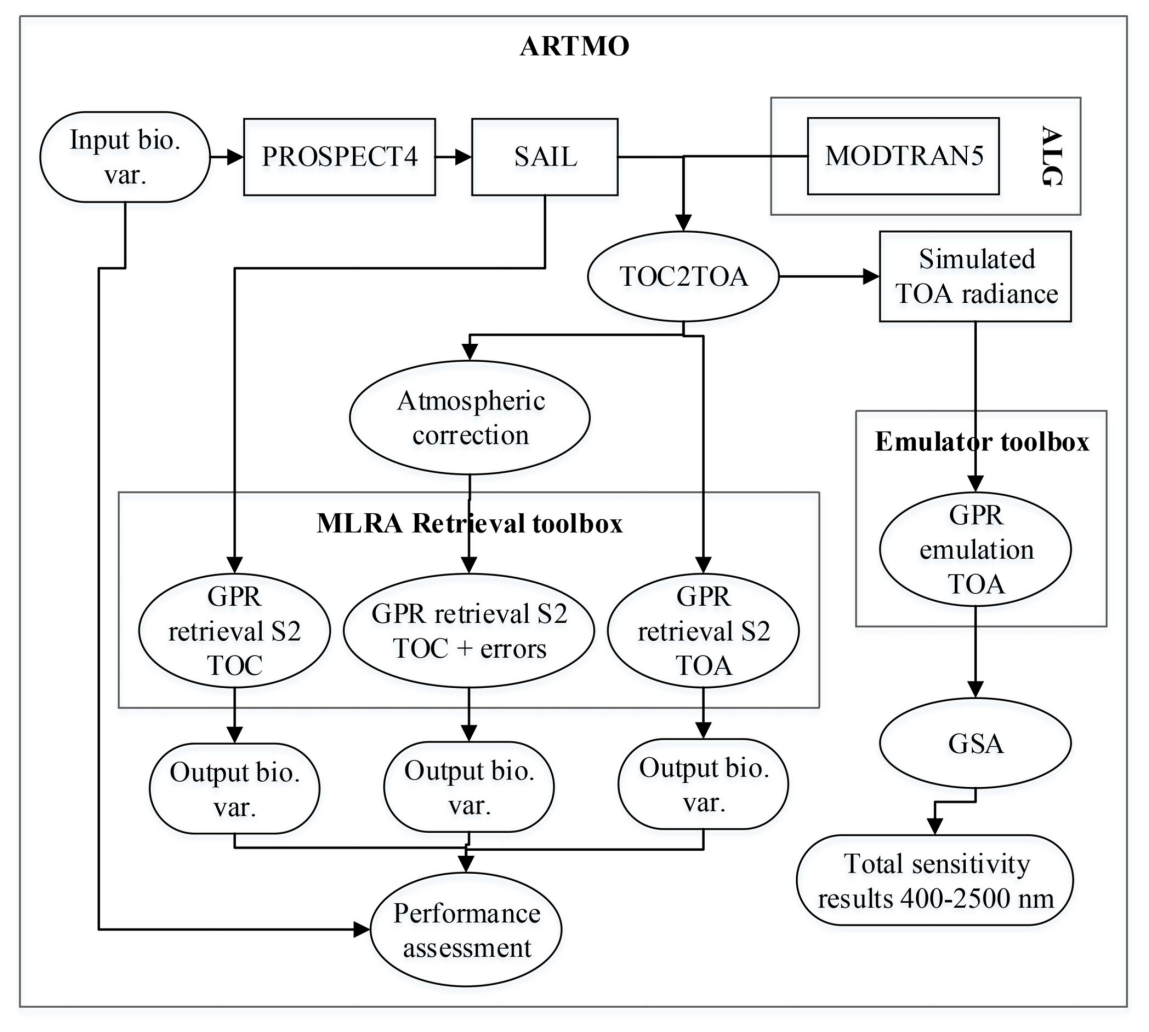
Flowchart of the pursued work-flow, divided into the Global Sensitivity Analysis (GSA) study (**right**) and retrieval performance assessment (**left**).

The general work flow is presented in Figure 2. In order to analyze the feasibility of retrieving biophysical variables from TOA radiance, we performed three parallel studies. First, a GSA using an emulator was carried out to determine the relative influence of each biophysical and atmospheric variable in the TOA radiance signal. Secondly, a set of synthetic test scenarios were generated to assess the performance of biophysical variables retrieval under controlled conditions. Three different retrieval scenarios were implemented: (1) ideal surface reflectance data (i.e., without errors from atmospheric correction), (2) TOA radiance, and (3) realistic surface reflectance data (i.e., affected by error propagation from atmospheric correction). Finally, retrieval strategies were applied to a real S2 data at L1C (TOA radiance) and L2A (BOA reflectance). A detailed description of the used processing tools and simulated datasets is provided in Sections 3.1 and 3.2. The used GSA and emulation algorithms are described in Sections 3.3 and 3.4. Further information about the implemented method for retrieving the various biophysical variables from the simulated data and evaluating their accuracy is described in Section 3.5. The method to assess the performance on a real S2 image is then described in Section 3.6.

### 3.1 Developed Toolboxes for Automated Processing

This work was conducted within the in-house developed ARTMO GUI framework. Automated Radiative Transfer Models Operator (ARTMO) [53] is a Matlab scientific software package that provides tools and toolboxes for running a suite of leaf, canopy and atmosphere RTMs and for post-processing applications such as retrieval. The toolboxes used in this work are briefly explained below.

- Atmospheric Look-up table Generator (ALG) [19] is an independent software tool that can be plugged into ARTMO and allows generating and analyzing LUTs based on a suite of atmospheric RTM, i.e., MODTRAN, 6SV, libRadtran.
- A new so-called “TOC2TOA” toolbox has been developed to enable coupling surface reflectance simulations with atmospheric simulations, i.e., to reach TOA radiance data. The TOC2TOA toolbox couples the atmospheric transfer functions with canopy reflectance simulations or observations to enable TOA radiance data, thereby ensuring that consistent geometry at canopy and atmosphere is preserved. Either canopy LUTs, surface reflectance data, e.g., from a field spectroradiometer, or a BOA reflectance image can be coupled with atmospheric transfer functions to enable uppscaling to TOA radiance data. In this version (1.0), the coupling assumes a Lambertian and homogeneous surface according to the formulation proposed in [54].
- The Global Sensitivity Analysis (GSA) toolbox [55] calculates a global sensitivity analysis on RTMs. The GSA toolbox enables to identify key driving input variables as well as non-influential input variables across the spectral range of spectral outputs. The main limitation of GSA is that it requires many simulations, and is thus limited by the processing speed of the model under study [32].
- To speed up GSA run-time, the GSA toolbox can be coupled with the Emulator toolbox [31,32]. This toolbox enables the evaluation of machine learning regression algorithms on their capability to approximate RTM outputs as a function of input variables.
- The machine learning regression algorithms (MLRA) toolbox [56] is one of ARTMO’s retrieval toolboxes. The MLRA toolbox contains over 20 MLRAs that can be trained and validated with either experimental or RTM data. Afterwards, a selected model can be applied to an image for mapping applications.

### 3.2 Description of Simulated Datasets

The training and performance assessment of biophysical parameters retrieval from at-sensor TOA radiance is based on simulated data of surface reflectance and TOA radiance. The use of RTMs allows us to test the retrieval accuracy under controlled conditions. On the one hand, surface reflectance datasets are based on the combination of PROSPECT-4 [20] and SAIL [21] RTMs, also known as PROSAIL. PROSPECT-4 is one of the most widely used RTMs that simulates leaf optical properties. The model calculates directional-hemispherical reflectance and transmittance measured from 400 nm to 2500 nm at 1 nm spectral sampling. SAIL solves the radiative transfer equation for scattering and absorption of four upward/downward fluxes at the canopy scale. In combination with PROSPECT-4 leaf optical properties, SAIL provides top-of-canopy (TOC) reflectance in the 400–2500 nm spectral range at 1 nm sampling. On the other hand, MODTRAN5 [24,57] was chosen to simulate the radiative transfer in the atmosphere at 15 cm^–1^ (0.3–9 nm in the covered spectral range of 400–2500 nm). MODTRAN has been extensively used for remote sensing applications such as atmospheric correction [6,7,58]. It solves the RT equation with an accurate simulation of the coupled absorption/emission and scattering effects by molecules and particulate matter in a multilayered spherically symmetric atmosphere [59,60]. With the application of the interrogation technique developed in [54], MODTRAN can generate the following output *atmospheric transfer functions:* Atmospheric path radiance (L_0_), direct/diffuse at-surface solar irradiance (*E_dir/dif_*), direct/diffuse target-to-sensor transmittance (*T_dir/dif_*), and spherical albedo (*S*).

**Figure 3:**
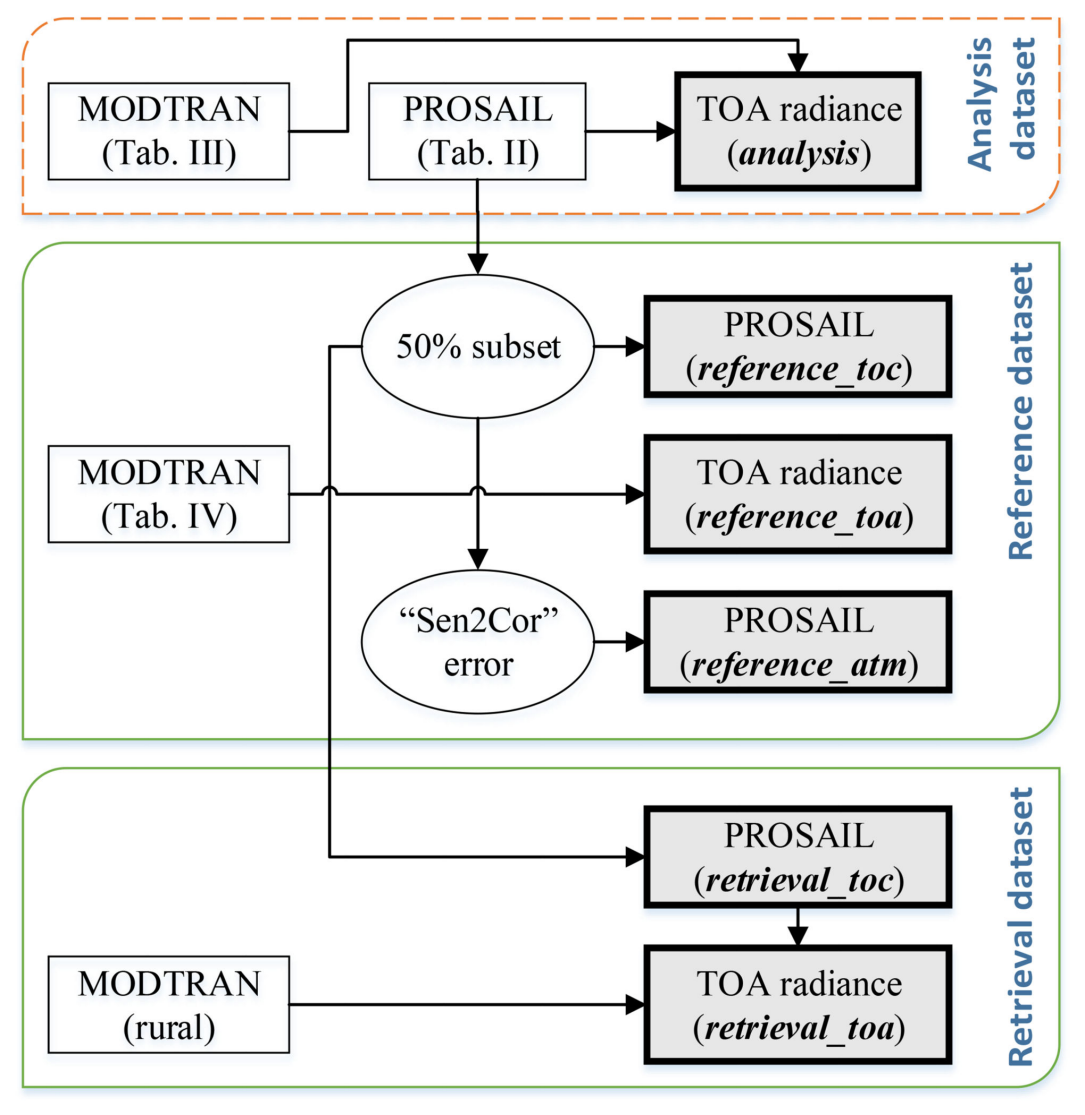
Schematic representation of synthetic datasets based on PROSAIL and MODTRAN simulations. The red-dashed and green lines identify the datasets used respectively for Global Sensitivity Analysis and Biophysical parameters retrieval.

The generation of the simulated datasets (*analysis, reference* and *retrieval)* is represented in Figure 3 and further described in the paragraphs below.

The first dataset (further referred to as *analysis*) functions to train an emulator that allows running a GSA to evaluate the relative contribution into TOA radiance of various leaf-canopy and atmospheric properties. Thus, this dataset combines PROSAIL and MODTRAN into a database of TOA radiance spectra. The first step was to generate the LUT of directional reflectance (*ρ*) derived from the combination of the PROSPECT-4 and SAIL. A set of 10,000 samples of the six input leaf-canopy variables were distributed according to a Latin Hypercube Sampling (LHS) distribution [61] (see Table 2). The hot-spot, soil brightness coefficient, and the sun-target senor geometry variables have been excluded from the analysis in order to facilitate the coupling between the vegetation and atmospheric RTMs. Simulations were carried out in the 400–2500 nm spectral range at 1 nm sampling.

The second step involves generating MODTRAN simulations. The *analysis* dataset contains 10,000 MODTRAN simulations sampled with a LHS distribution (see Table 3). These simulations were carried out in the same spectral range as PROSAIL simulations with a sampling of 15 cm^–1^ (0.3–9 nm in the covered spectral range of 400–2500 nm). Input variables were selected so that they have an impact in the entire wavelength range (400–2500 nm) and include typical variability [8,62–64] in: (1) the AOT550; (2) the spectral dependency of the extinction coefficient, through the Ångström exponent; (3) the phase function, through the HG asymmetry parameter; (4) the single scattering albedo; and column-integrated concentrations of (5) ozone (O3C) and (6) vapor (CWV).

The surface (PROSAIL) and the atmospheric (MODTRAN) simulations were randomly one-to-one combined (10’000 simulations) and propagated to TOA radiance following Equation (1) with the Lambertian and homogeneous surface assumption:

(1) $$L=L_{0}+\frac{E_{tot}T_{tot\;}\rho }{\pi (1-S\rho )}$$

where *T_tot_ = T_dir_* + *T_dif_* is the total target-to-sensor transmittance and *T_tot_* = *E_dir_* cos *θ_il_* + *E_dif_* is the total at-surface irradiance for a solar zenith angle *θ_il_*. Here, the 1 nm sampling surface reflectance (*ρ*) was interpolated by cubic splines to the MODTRAN wavelength grid. For the sake of simplicity, the spectral dependency of all terms in the Equation (1) has been omitted. A random subset of 1000 cases is then used to train an emulator for further GSA calculation.

The second dataset (further referred to as *reference*) aims at representing realistic S2 observations over land surfaces for broad atmospheric conditions and is used for validation. The *reference* dataset is divided into three subsets to validate the retrieval strategies under three different scenarios (see Figure 3): (1) retrieval from an ideal surface reflectance data, (2) retrieval from TOA radiance, and (3) retrieval from surface reflectance after a non-perfect atmospheric correction. The first subset (*reference_toc*) corresponds to a reference surface reflectance dataset composed of a random subset of 5000 samples extracted from the *analysis* dataset previously described in Table 2. This scenario should be taken as the ideal case, since there are no radiometric perturbances due to atmospheric scattering and absorption. This surface reflectance data is combined with other 5000 MODTRAN simulations to create the second subset of reference TOA radiance (*reference_toa)*. This second scenario refers to the goal of this paper i.e., to validate the performance of retrieving biophysical variables directly from TOA radiance. In this case, MODTRAN simulations were run with varying conditions of CWV, O3C, AOT_550_ and aerosol type with an LHS distribution (see Table 4) and in the same spectral range and sampling as in the *analysis* dataset. With respect the aerosol type, the following 9 models were included: MODTRAN’s rural, urban and navy-maritime (with 3 air mass values identifying coastal to strong land influence), and OPAC’s continental (clean, average and polluted) and urban [62].

Both the *reference_toc* and the *reference_toa* subsets were convolved by S2 instrument spectral response function (ISRF, *f_c_*) for each spectral channel *c* (1 to 13) [42] following Equation (2):

(2) $$L_{c}=\frac{\int L\cdot f_{c}d\lambda }{\int f_{c}d\lambda }$$

**Figure 4:**
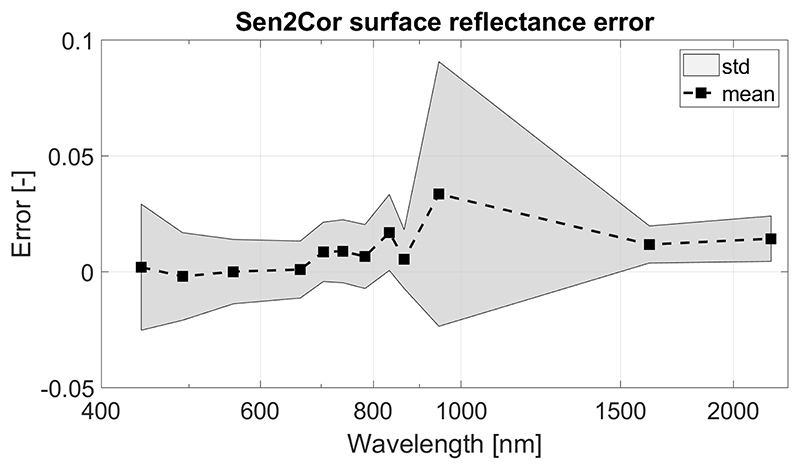
Typical error in surface reflectance after Sen2Cor atmospheric correction algorithm. See Table VIII in [50] for detailed information.

The third subset (*reference_atm)* aims to represent surface reflectance spectra obtained after a non-perfect atmospheric correction as would be from Sen2Cor algorithm. Instead of implementing an atmospheric correction process on the *reference_toa* subset, the *reference_toc* subset was perturbed in order to reproduce the expected error propagation from the Sen2Cor algorithm [50]. Accordingly, the *reference_atm* surface reflectance spectra (*p_atm_*) is created from the *reference_toc* spectra (*ρ_toc_*) following Equation (3):

(3) $$\rho_{atm}=\rho_{toc}+\varepsilon_{\rho }$$

where *ε_ρ_* is the expected wavelength-dependent error from Sen2Cor shown in Figure 4.

Finally, the third dataset (further referred to as *retrieval*) is used to train the retrieval algorithms for each of the biophysical variables (see Section 3.5). The *retrieval* dataset consists of two subsets of surface reflectance (*retrieval_toc*) and TOA radiance (*retrieval_toa*), both generated with the same process as for the construction of the *reference* dataset. Regarding the *retrieval_toc* subset, this is constructed from the remaining 50% samples from the *analysis* dataset that were not used in the *reference_toc* subset. The TOA radiance subset uses a new set of 5000 MODTRAN simulations with the same input variables as in Table 4 but only using MODTRAN’s rural aerosol type. In this way, the retrieval of biophysical variables from TOA will carry along errors due to uncertainties in aerosol optical properties.

### 3.3 Global Sensitivity Analysis (GSA)

In order to identify the driving vegetation and atmospheric variables having an impact on TOA radiance, we first conducted a global sensitivity analysis (GSA) of the TOA radiance simulations from the *analysis* dataset. Most GSA methods are variance-based methods, which decomposes the variance of the model output into fractions that can be attributed to inputs or sets of inputs [28,65]. While the Sobol’ method [66] pioneered in developing a variance-based GSA method, a modified version was proposed by [67], which proved to be effective in identifying the so-called Sobol’s sensitivity indices. These indices quantify both the main sensitivity effects (first-order effects: *S_i_*, i.e., the contribution to the variance of the model output by each input variables) and total sensitivity effects (*S_Ti_*, i.e., the first-order effect plus interactions with other input variables) of input variables. This method has been applied here. A description according to [68] is given below.

Formally, we have a model *y* = *f* (**x**), where *y* is the model output, and **x** = [*x*_1_, *x*_2_,..., *x_k_*]^T^ is the input feature vector. A variance decomposition of *f* (·) as suggested by Sobol [66] is:

(4) $$V(y)=\sum_{i=1}^{k}V_{i}+\sum_{i=1}^{k}\sum_{j=i+1}^{k}V_{ij\ldots }+V_{1,\ldots ,k\prime }$$

where **x** is rescaled to a *k*-dimensional unit hypercube ${\text{Ω}}^{k},{\text{Ω}}^{k}=\left\{x|0\leq x_{i}\leq 1,i=1,\ldots ,k\right\}$; 𝕍(*y*) is the total unconditional variance; *V_i_* is the partial variance or ‘main effect’ of *x_i_* on *y* and given by the variance of the conditional expectation *V_i_* = 𝕍[𝔼(*y*|*x*_i_)]; *V_ij_* is the joint impact of *x_i_* and *x_j_* on the total variance minus their first-order effects. Here, the first-order sensitivity index *S_i_* and total effect sensitivity index *S_Ti_* are given as [28]:

(5) $$S_{i}=\frac{V_{i}}{V(y)}=\frac{V\left[E\left(y|x_{i}\right)\right]}{V(y)}$$

and:

(6) $$S_{Ti}=S_{i}+\sum_{j\neq i}S_{ij}+\ldots =\frac{E\left[V\left(y|x_{∼i}\right)\right]}{V(y)},$$

where *x_~i_*, denotes variation in all input variables and *x_i_*, *S_ij_* is the contribution to the total variance by the interactions between variables. Following Saltelli et al. (2010) [67], to compute *S_i_* and *S_Ti_*, two independent input variable sampling matrices **P** and **Q** of dimensions *N × k* are created, where *N* is the sample size and *k* is the number of input variables. Each row in matrices **P** and **Q** represents a possible value of **x**. The variable ranges in the matrices are scaled between 0 and 1. The Monte Carlo approximations for 𝕍(*y*), *S_i_* and *S_Ti_* are defined as follows [67,69]:

(7) $$\hat{V}(y)=\frac{1}{N}\sum_{j=1}^{N}{\left(f{\left(P\right)}_{j}\right)}^{2}-{\hat{f}}_{0}^{2},\;{\hat{f}}_{0}=\frac{1}{N}\sum_{j=1}^{N}f(P{)}_{j},$$

and:

(8) $$\hat{S_{i}}=\frac{1}{N}\sum_{j=1}^{N}\frac{f{\left(Q\right)}_{j}\left(f{\left(P_{Q}^{\left(i\right)}\right)}_{j}-f{\left(P\right)}_{j}\right)}{\hat{V}(y)}$$

and:

(9) $$\hat{S_{Ti}}=\frac{1}{2N}\sum_{j=1}^{N}\frac{{\left(f{\left(P\right)}_{j}-f{\left(P_{Q}^{\left(i\right)}\right)}_{j}\right)}^{2}}{\hat{V}(y)},$$

where $\hat{\ldots }$ is the estimate; ${\hat{f}}_{0}$ is the estimated value of the model’s output; we abused notation by defining *f* (**P**) as all outputs for row vectors in **P**; $P_{Q}^{\left(i\right)}$ represents all columns from **P** except the *i^th^* column which is from **Q**, using a radial sampling scheme [70]. Matrices are generated with an LHS of size *N* × 2*k* where **P** and **Q** are the left and right half of this matrix, respectively [67]. In order to compute *S_i_* and *S_Ti_* simultaneously, a scheme proposed by [29] was used, which reduced the model runs to *N*(*k*+2).

### 3.4 Emulation

Instead of entering the computationally expensive coupled PROSAIL-MODTRAN into GSA, we used an emulated version of these coupled models. Emulation is a statistical learning technique used to estimate model simulations when the model under investigation is too computationally costly to be run many times [71]. The basic idea is that an emulator uses a limited number of simulator runs, i.e., input-output pairs (corresponding to training samples), to train a machine learning regression algorithm (MLRA) in order to infer the values of the complex simulator output given a yet-unseen input configuration. These training data pairs should ideally cover the multidimensional input parameter space using a space-filling sampling algorithm, e.g., LHS. Once the emulator is built, it is not necessary to perform any additional runs of the model; the emulator computes the output that is otherwise generated by the RTM.

When it comes to emulating RTM spectral outputs, however, the challenge lies in delivering a full spectrum. This implies that the MLRA should be able to generate multiple outputs to reconstruct a full spectral profile, which is not a trivial task. For instance, the contiguous spectral profile between 400 and 2500 nm consists of over 2000 bands when binned to 1 nm resolution. Only some MLRAs can obtain multi-output models, but that typically lead to highly complex models with long training time and certain risk of overfitting because of model over-representation, e.g., as with neural networks. A workaround solution was developed that enables the regression algorithms to cope with large spectroscopy datasets by taking advantage of the so-called *curse of spectral redundancy*, i.e., the Hughes phenomenon [72]. Since spectroscopy data usually shows a great deal of collinearity, it implies that such data can be compressed to a lower-dimensional space through dimensionality reduction techniques such as principal component analysis (PCA) [73]. Accordingly, spectroscopy data can be converted into components, which are only a fraction of the original amount of bands, and implies that the multi-output problem is greatly reduced to a number of components that preserve the spectral information content (see also [74–77]). Afterwards, the components are then reconstructed again to spectral data [31–33,36,77].

In earlier RTM emulation evaluation studies [31–33], various MLRAs were analyzed on their predictive performance. In each of these studies Gaussian processes regression (GPR) [78] was evaluated as the top performing one. Although its superior performance went somewhat at the expense of processing speed as opposed to other MLRAs, it runs numerous times faster than the original RTM [31–33]. GPR is a probabilistic kernel method, and has been widely used for retrieval of biogeophysical variables and emulation applications [79–81]. Kernel methods in machine learning owe their name to the use of kernel functions [82–84]. These functions quantify similarities between input samples of a dataset. Similarity reproduces a linear dot product (scalar) computed in a possibly higher dimensional feature space, yet without ever computing the data location in the feature space.

GPR generalize Gaussian probability distributions in function spaces [78]. The prediction and the predictive variance of the model for new samples are given by:

(10) $$\hat{f}\left(x_{q}\right)=\sum_{i=1}^{n}ik\left(x_{i},x_{q}\right)$$

(11) $$V\left[\hat{f}\left(x_{q}\right)\right]=k\left(x_{q},x_{q}\right){-}_{*}^{⊤}{\left(K+\sigma_{n}^{2}I\right)}_{*}^{-1}$$

where *k*(·, ·) is a covariance (or kernel function), * is the vector of covariances between the query point, **x**_*q*_, and the *n* or training points, and $\sigma_{n}^{2}$ accounts for the noise in the training samples. As one can see, the prediction is obtained as a linear combination of weighted kernel (covariance) functions, the optimal weights given by $w={\left(K+\sigma_{n}^{2}I\right)}^{-1}f(x)$. Many different functions can be used as kernels for [85]. We used the automatic relevance determination squared exponential kernel for GPR, which has a separate length hyperparameter for each input dimension. Stochastic gradient descent algorithms maximizing the marginal log-likelihood are employed, which allow optimizing a large number of hyperparemeters in a computational effective way.

Based on experience from earlier emulation exercises [32,33], the TOA radiance data was first compressed into 20 PCA components. The GPR emulator was trained with 70% of the 1000 samples and the remaining dataset was kept for validation. Goodness-of-fit statistics were calculated to assess the emulator’s capability to generate accurate TOA radiance data: the Pearson’s correlation coefficient (*R*^2^) and root-mean-square error (RMSE) are calculated according to Equations (12) and (13):

(12) $$R^{2}=\frac{n\sum_{i=1}^{n}\left(X_{ref,i}\cdot X_{ret,i}\right)-\sum_{i=1}^{n}X_{ref,i}\cdot \sum_{i=1}^{n}X_{ret,i}}{\sqrt{\left[n\sum_{i=1}^{n}X_{ref,i}^{2}-{\left(\sum_{i=1}^{n}X_{ref,i}\right)}^{2}\right]\left[n\sum_{i=1}^{n}X_{ret,i}^{2}-{\left(\sum_{i=1}^{n}X_{ret,i}\right)}^{2}\right]}},$$

and:

(13) $$RMSE=\sqrt{\frac{1}{n}\sum_{i=1}^{n}{\left(X_{ref,i}-X_{ret,i}\right)}^{2}},$$

where *X_ref,i_*, and *X_ret,i_* are respectively the reference and retrieved values.

Finally, the trained GPR emulator was imported into the GSA toolbox. In the GSA toolbox, the number of emulations executed was (N(k+2), where N represents the number of samples and k the number of input variables. We chose 1000 runnings per variables. This led to 14,000 runnings to compute the GSA sensitivity indices. The GSA results provide insights into the role of the driving variables at TOA as observed by a satellite sensor. Based on these insights, hybrid retrieval schemes were developed for retrievable biophysical variables, as described below.

### 3.5 Hybrid Retrieval Schemes

When it comes to selecting a biophysical variable retrieval method for processing large images such as Sentinel-2 (S2), it requires models that are fast, robust and easily applicable. Based on a systematic comparison of parametric, non-parametric and RTM-inversion retrieval methods taking both accuracies and run-time into account [86], it was concluded that hybrid retrieval schemes, i.e., machine learning methods trained by RTM simulations, can achieve both accurate and fast estimates. Regarding the used MLRA, similar as in emulation, GPR was evaluated as a powerful method for mapping applications [38,39,87]. Starting with Equation (10), we used a scaled Gaussian kernel function:

(14) $$k\left(x_{i},x_{j}\right)=v\exp\left(-\sum_{b=1}^{B}\frac{{\left(x_{i}^{\left(b\right)}-x_{j}^{\left(b\right)}\right)}^{2}}{2\sigma_{b}^{2}}\right)+\delta_{ij}\cdot \sigma_{n}^{2},$$

Regarding retrieval, three important properties of the method are worth stressing here. First, the obtained weights *w* after optimization gives the relevance of each spectrum **x***_i_* (see [88] for extended equations). The predictive mean is essentially a weighted average of the vegetation biophysical parameter values associated with the training samples closest to the test sample. Second, the inverse of *σ_b_*, represents the relevance of band *b*. Intuitively, high values of *σ_b_*, mean that relations largely extend along that band hence suggesting a lower informative content. These features have been extensively studied in [87,88] and proved to be valuable for gaining insight into relevant bands. Third, and particularly of interest for mapping applications, a GPR model provides not only a per-pixel prediction, but also an uncertainty (or confidence) level for the prediction. Hence, uncertainty intervals are directly delivered along with the variable estimates, which enables to assess the model transferability in space and time [86,88].

We assessed the performance on biophysical variable retrieval on the three reference scenarios previously described in Section 3.2. The MLRA retrieval toolbox was first used to train and to validate an MRLA from the *retrieval* datasets and then to apply the trained model to retrieve biophysical variables from the *reference* datasets. Based on experience from earlier retrieval exercises [32,33], the two *retrieval* databases were split into 70% for the training and 30% for the validation of the GPR retrieval algorithms. The *retrieval_toc* dataset was used to train and validate one GPR model for the retrieval of biophysical variables from surface reflectance. The *retrieval_toa* was instead used for the retrieval from TOA radiance. Goodness-of-fit statistics were calculated to assess the GPR models’ capability to retrieve accurately biophysical variables. The error difference between the reference and the retrieved biophysical variables is calculated for each of the 5000 samples in the *reference* database and the histogram plotted. In addition, Pearson’s correlation coefficient (*R*^2^) and root-mean-square error (RMSE) are calculated.

### 3.6 Retrieval of Biophysical Variables from Sentinel-2 L1C and L2A Images

As a proof-of-concept of the developed TOA radiance retrieval algorithms, a S2-A image was selected for both the TOA L1C and BOA L2A reflectance products. The chosen image was acquired by S2A on 22 August 2018 at 12:56 h (UTC time +2 h) over the area of Barrax (Spain). Barrax is a sparsely vegetated site located in Spain between 38.75°N and 39.75°N and 1.73°W and 3.00°W. It is predominately flat with a mean elevation of 700 m above sea level (a.s.l.), although there is some rugged terrain in the northeast reaching 1185 m a.s.l. For the given location and acquisition date, The image was illuminated with a mean SZA of 30.8°. Since the focus here is retrieval over vegetated surfaces, a subset over the Barrax agroecosystem was chosen (600 × 600 pixels). This region is characterized by large agricultural fields with center pivot irrigation systems. Main crops are wheat, alfalfa, rapeseed, sunflower and garlic. In August, the non-irrigated areas are bare soil or senescent vegetation. In addition, an AOT at 550 nm of approximately 0.15 was determined from the AERONET stations of Aras de los Olmos (at 130 km north-east) and Murcia (100 km south-east) at the time of observation.

As described in the section above (Section 3.5), we applied the GPR retrieval algorithms to S2 L1C and L2A data for the GSA-identified dominant thus retrievable variables. To do so, the S2 L1C TOA reflectance data first had to be converted to TOA radiance data, which is done in the SNAP toolbox. In addition, only the 10 m and 20 m bands were used at 20 m resolution without the broadband B8 as it is overlapping with B8a (see Figure 1).

Further, in an attempt to make the models better fit to process real S2 data, Gaussian noise was added to the *retrieval* TOC and TOA training datasets. The addition of noise to the RTM generated spectral bands has multiple purposes: it simulates errors of radiometric calibration, atmospheric noise and residuals from the atmospheric correction, but to some extent also bridge between the simplified representation of the RTM and the actual radiometric behaviour of the canopy [89]. Generally, noise prevents the retrieval model from over-fitting on the training database. However, an accurate quantification of all error terms in the sensing process remains difficult [89]. While for the TOC training dataset noise levels can be obtained from S2 surface reflectance studies as in [50], for TOA that is not the case. After some testing of additive and multiplicative noise levels, eventually, a 2% multiplicative Gaussian noise was used. The GPR model development and image processing were done in the MLRA toolbox. Finally, to account for the non-vegetated surfaces, 20 distinct bare soil spectral signatures were added to the L1C and L2A training datasets.

## 4 Results

Following the method described in Section 3, we show the results corresponding to: (1) the conducted GSA of the leaf-canopy-atmosphere RTM (Section 4.1), (2) the performance assessment on the retrieval of biophysical variables from synthetic S2 surface reflectance and TOA radiance (Section 4.2), and (3) the proof-of-concept results for the retrieval of biophysical variables from real S2 L1C and L2A data.

### 4.1 Global Sensitivity Analysis Results

A GPR emulator was first developed as approximation of the coupled PROSPECT4-SAIL-MODTRAN model given 12 input variables and 1000 samples taken from the *analysis* dataset. GPR was used because of superior performances as opposed to other MLRAs [31–33]. This was also the case here: GPR clearly outperformed competing algorithms such as neural networks and kernel ridge regression (results not shown). Training the GPR emulator took less than 3 min. It reached an overall accuracy with RMS errors (RMSE) of 1.06 and normalized RMS errors (NRMSE) of 2.39%. When plotting the NRMSE along the spectral range, it appears that accuracies are consistent with the exception of the water absorption regions around 1400 and 1900 nm where accuracies are somewhat poorer (errors around 6%) (results not shown). This accuracy is on the same order as earlier published emulators [33,90], and can be considered as adequate for subsequent GSA calculations.

**Figure 5:**
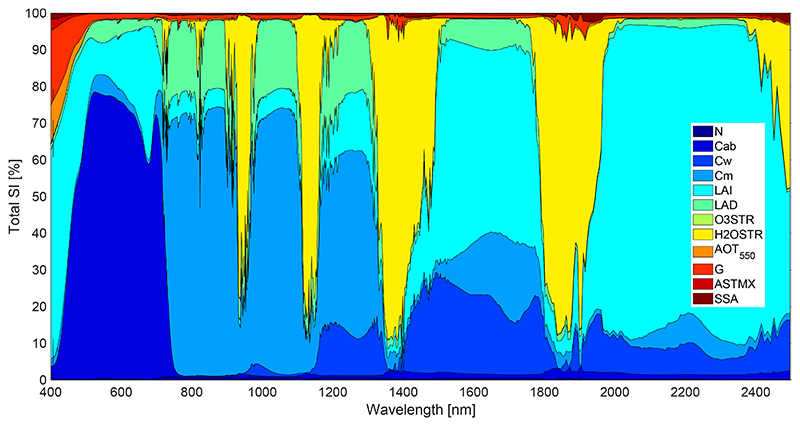
Total sensitivity (*S_Ti_*) results of TOA radiance using a GPR emulator of a 12 variables PROSAIL-MODTRAN model. See Tables 2 and 3 for the full names of the variables.

Running the GPR emulator into the GSA with 1000 samples per variable took less than 40 s. In comparison, if the same analysis would have been done by the original coupled RTMs, run-time would take on the order of several weeks. The total sensitivity GSA results shown in Figure 5 are expressed as relative contributions to output variance for each one of the input variables in the TOA spectrum (*S_Ti_*, expressed in %). The figure leads to the following observations:

- Generally, the GSA results indicate that atmospheric variables had a weaker influence than vegetation variables. Regarding the atmospheric variables, clearly, the H_2_O content had a strong impact in discrete parts of the spectrum, in agreement with the location of H_2_O absorption bands. Relatively small impact bands can be found at 820 nm, while stronger impact (over 70% *S_Ti_*) in the region of 900–950 nm and 1100–1150 and the largest impact bands (over 80%*S_Ti_*) between 1350–1450 and between 1800–1900 nm where the H_2_O absorption saturates.
- The aerosol optical properties (extinction, absorption and phase function) were the most dominant atmospheric variables. Particularly, the AOT_550_ and phase function (through the Henyey-Greenstein parameter, G) had a relatively strong impact (30%*S_Ti_)* in the region of 400 to 500 nm, where the scattering is higher. This impact diminishes to a few percents in the range of 500 to 1300 nm and with barely any influence after 1300 nm. According to the GSA results, the O_3_ seemed not to have a relevant influence over the variance of the TOA radiance even at the bottom of the Chappuis absorption band (400–650 nm) where the O_3_ absorption is higher.
- Among vegetation variables, at the leaf level, chlorophyll content (Cab) was the main driver of TOA radiance in the visible range (450–750 nm) with over 60% *S_Ti_*, while dry matter content (Cm) was the main driver in the NIR range (750 to 1200 nm), 70%. Water content (Cw) had a negligible impact on the visible and the NIR but had a considerable impact in the SWIR (1400 to 2500 nm), with *S_Ti_* up to 20%. These three variables explain more than the 60% of the variance along the visible and NIR spectral range (400–1400 nm). The leaf layer variable (N) had a rather weak influence, but it covered the whole spectral range. Among canopy variables, LAI is the most dominant variable. It has influence along the whole spectral range, but it becomes especially dominant from 1400 nm onwards. LAI especially dominates the 2000–2400 nm SWIR region with a *S_Ti_* of around 80%.

From a practical point of view, the GSA results suggest that it should be perfectly possible to retrieve Cab (dominant in the visible), Cw (dominant in the NIR-SWIR) and LAI (dominant in the NIR-SWIR) from TOA radiance data. When interpreting these results in view of S2 band settings (see Figure 1), we observed that atmosphere has little influence in the SWIR, B11 at 1610 nm and B12 at 2190 nm. These bands seem to be particularly appropriate for LAI retrieval. At the same time, given the dominance of Cab in the visible, and the relatively strong contribution of Cw further in the NIR-SWIR; these regions are well covered by S2 bands. It is therefore worthwhile to explore the retrievability of these three biophysical variables directly from S2 TOA radiance data.

### 4.2 Biophysical Variables Retrieval

GPR models were developed for the variables Cab, Cw and LAI using the *retrieval* training data with noise added for nine bands at 20 m (without B8). These models were then validated against simulated *reference* data for: (1) the TOC scale, (2) TOA reference data, and (3) TOC dataset with noises according to Sen2Cor atmospheric correction errors. At the TOC scale, it was no surprise that retrievals against the TOC reference dataset led to excellent validation results with for Cab and Cw an R^2^ of 0.94–0.97 (Table 5). LAI was poorer validated, with a R^2^ of 0.68, due to saturation for higher LAI values, i.e., above 3. This suggests that the LAI model is suboptimally trained. What is more of importance in the context of this study is that results only degrade slightly when upscaling to TOA data, thus with the inclusion of atmospheric variables in the LUT. Excellent results are again obtained for Cab and Cw (R^2^ of 0.91–0.95), while poorer yet consistent results are achieved for LAI (R^2^ of 0.62). When moving back to the TOC scale, but now with adding noise levels according to Sen2Cor atmospheric correction errors, the results tend to degrade further. Given that this exercise is conducted with simulated data, the latter scenario is considered closer to reality. Comparison of these results may suggest that retrieving biophysical variables directly from TOA radiance data can be more beneficial, however, that is yet to be evaluated when applying to real data.

**Figure 6:**
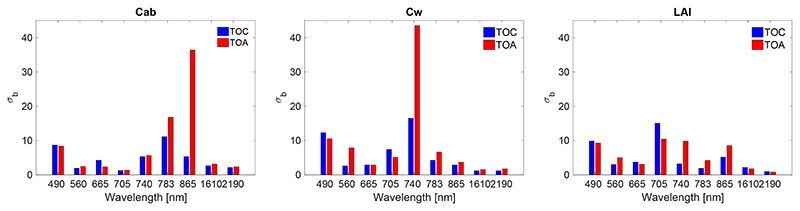
GPR band sigma (*σ_b_*) for trained models for S2 L2A TOC and L1C TOA data. The lower the *σ_b_*, the more important the band in the model development.

An easy way to gain insight into the functioning of the GPR models at TOC and TOA scale is by means of inspecting the sigmas (*σ_b_*), i.e., the band relevance, of the trained GPR models. They have been plotted in Figure 6 for the three biophysical variables. The lower the *σ_b_* is, the more important the band. Accordingly, the *σ_b_* reveal the most important wavelengths with spectral information used for the development of the models. The following observations can be made:

- Overall, no systematic differences between TOC and TOA *σ_b_* can be observed. About the same patterns appeared with low *σ_b_* for the majority of bands. This suggests that models can be developed from both TOC and TOA data sources with about the same degree of retrieval success.
- A closer inspection towards Cab and LAI reveals that TOA data led to considerably higher *σ_b_* for some bands (i.e., 490, 783 and 865 nm for Cab; 490 and 740 nm for Cw). This suggests that for these variables the TOA data has more difficulty to develop the retrieval algorithms. Conversely, the *σ_b_* similarity between the TOC and TOA bands for the LAI models suggests that the role of atmosphere is of marginal importance for LAI retrieval.
- For all variables, the band in the blue is evaluated as poorly contributing, both for TOC and TOA. For TOA this can be explained by the influence of aerosols, while at TOC scale this may be rather due to the remaining impact of the aerosols in the atmospheric correction. It is also of interest that the SWIR bands play an important role for TOC and TOA retrieval algorithms.

**Figure 7:**
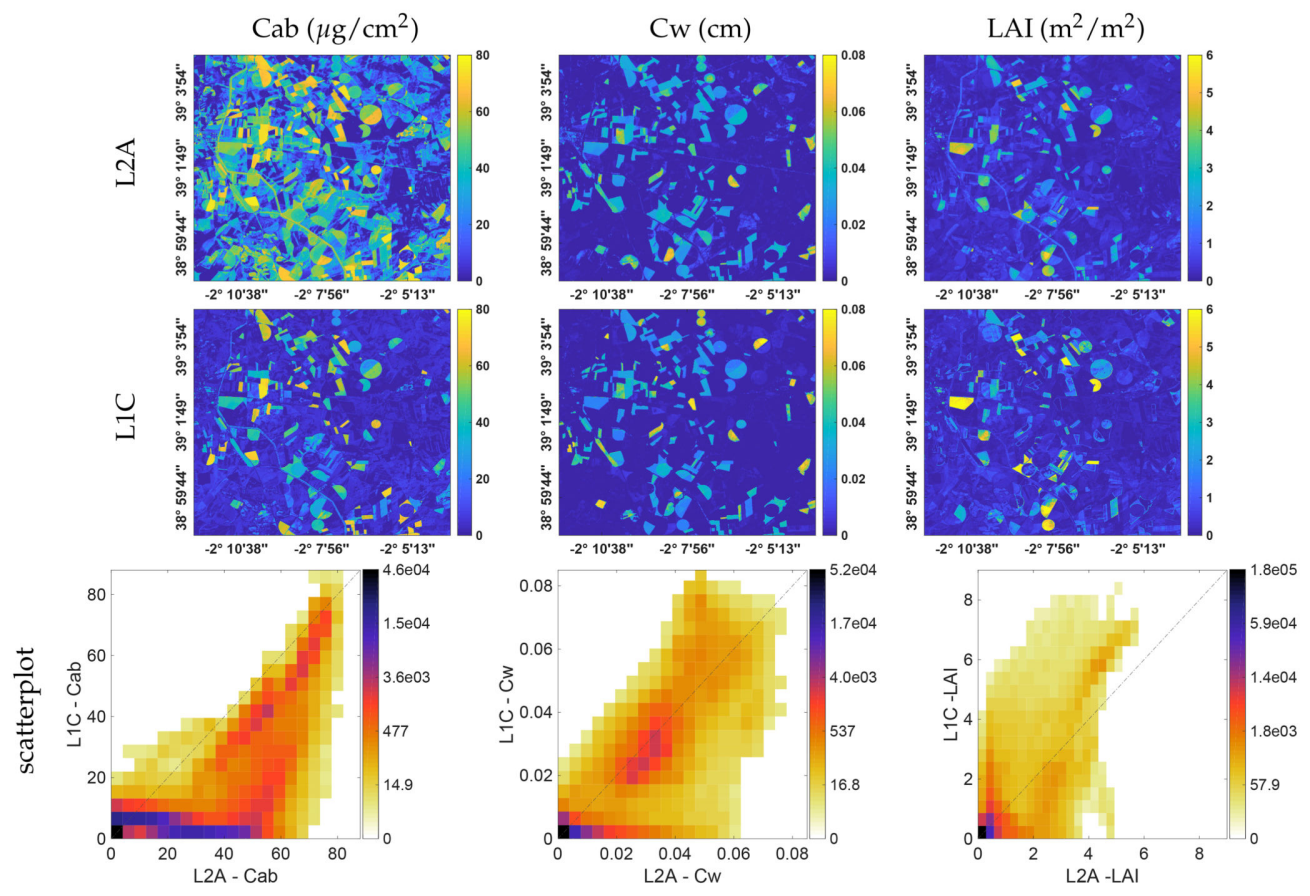
Maps of biophysical variables obtained from L2A (**Top**) and L1C (**Middle**) data. Scatter plots of both maps with gridded color density (**Bottom**).

Finally, as a demonstration case, we applied the TOC- and TOA-trained GPR models to a cloud-free subset of a S2 L1C and L2A imagery over the Barrax region to evaluate the actual performance of the models to convert S2 data into maps. The obtained maps are shown in Figure 7. At a glance, the similarity between both L1C and L2A maps can be observed; for both data levels, reasonable retrievals are obtained. This is encouraging, as it suggests that retrievals can be directly obtained from L1C data given a cloud-free sky, but a closer inspection is necessary to evaluate the quality of both products. Clearly differences appear. For Cab, the L2A product provides a sharper contrast between vegetated and non-vegetated surfaces with probably some overestimations. The Cw map looks most similar, while LAI is generally underestimated with probably overestimation for L1C over vegetated surfaces. These differences are also reflected in the scatter plots shown underneath. They indicate that, despite some mismatch for non-vegetated surfaces, Cab and Cw perform alike, for Cab a systematic overestimation for the L2A product as compared to the L1C product. On the other hand, the LAI L1C and L2A products are more poorly correlated; particularly the L1C yielded considerably higher estimates over the green irrigated areas. Yet, it must be remarked that both LAI models require improvements. This and other limitations are discussed further on.

## 5 Discussion

Here we discuss the various processing steps that are required to achieve a generic TOA retrieval processing chain, i.e., (1) emulation, (2) GSA, and (3) retrieval. These steps have been streamlined and automated thanks to the development of some dedicated toolboxes. We will therefore close the discussion with prospects for further improvements.

### 5.1 Emulation of Leaf-Canopy-Atmosphere RTMs

The first objective was to identify the driving variables of vegetated surfaces that shaped the TOA radiance reaching an optical sensor in space. To do so, RTMs of leaf, canopy and atmosphere were coupled. The coupling process of the leaf-canopy-atmosphere RTMs-PROSPECT-4, SAIL and MODTRAN-allowed to simulate LUT of TOA radiance data assuming a Lambertian surface. However, because MODTRAN is computationally expensive and takes some seconds to run a single simulation, it implies that running thousands simulations can take days to weeks. To overcome this computational burden, with emulation a bypass was found to speed up the production of simulated TOA radiance data [32]. Given the assumption that the emulator approximates the TOA radiance outputs of the original leaf-canopy-atmosphere RTMs with sufficient accuracy, it can then be safely used for RTM-based applications such as GSA studies. By having the emulator producing the simulations quasi instantly, the GSA was processed in the order of seconds. Validation against reference data showed that the emulator can reproduce TOA radiance with sufficient accuracy (NRMSE errors of 2.4%) for conducting reliable GSA studies [91]. The emulation accuracy is consistent with earlier analysis for the emulation of PROSPECT-4, PROSAIL and MODTRAN [90]. Based on this and earlier studies, the following observations can be drawn. The accuracy of the emulator depends on the type of the algorithm used, number of variables, number of training samples and complexity of the model [32,90,91]. From multiple machine learning methods tested such as neural networks and other kernel-based methods, in this (results not shown) and earlier studies, Gaussian processes regression (GPR) yielded the highest accuracies in approximating the outputs of the original RTM. Thereby, accuracies can be improved with adding more training data although that is at the expense of processing speed. Typically, training with about 1000 simulations is considered as a good trade-off between accuracy and optimizing processing speed, e.g., for GSA calculations [33,90].

### 5.2 GSA

Although earlier sensitivity studies have studied aspects of coupled leaf-canopy-atmosphere models along the spectral range e.g., [16,18,92], these are “local” studies sensitivity studies, i.e., keeping the majority of variables fixed. With “global” sensitivity analysis (GSA), all variables are ranged at the same time and interactions are calculated. Variance-based GSA proved to be a powerful tool to determine and study the main drivers that govern TOA radiance as observed by a sensor. This becomes extremely relevant in identifying retrievable biophysical variables [2,93]. In principle, a high sensitivity value indicates that the input variable is responsible for a significant portion of the output variance and should thus be possible to retrieve e.g., as recorded by an Earth observing satellite. To the best of our knowledge, this is the first time that a GSA was used to decompose the full leaf-canopy-atmosphere radiative transfer of TOA radiance into their driving variables along the 400–2500 nm spectral range at 1 nm resolution. The most remarkable GSA result is the relatively small contribution of the atmospheric variables driving the TOA radiance variance. This indicates that the contribution of vegetation variables is much more important than the contribution of atmospheric variables. In view of mapping applications, it implies that the retrieval of biophysical parameters from TOA radiance should be certainly possible. Moreover, small inaccuracies in the atmospheric data do not affect the sensitivity of the vegetation variable in the TOA radiance [11]. Regarding the atmosphere drivers, H_2_O concentration is the most dominant variable, but it only appears in the water vapor absorption bands; its presence outside these bands appeared negligible as opposed to other drivers. In general, optical sensors do not consider these water vapor absorption bands for biophysical variable retrieval. O_3_ concentration has no effect in the GSA, neither at the Chappuis band (400–650 nm), where O3 has it absorption bands. Outside the water vapour absorption bands, aerosol optical thickness (AOT) is the atmospheric variable with the strongest impact on TOA radiance. Its importance is especially relevant at the lower part of the spectrum (400–500 nm) as combination of high aerosol absorption/scattering and low surface reflectance. This suggests that the retrieval of biophysical parameters would be more feasible in clear atmospheric days and further away in the spectrum. These results can be related to the ones found by [11,93], who observed that the sensitivities of surface reflectance are comparable to the TOA radiance sensitivities, which implies that atmospheric variables have a rather weak influence in driving variability of the TOA radiance data. Obviously, this is only valid given a cloud-free sky.

Both leaf and canopy variables drive the TOA radiance along the 400–2500 nm spectral range outside the water vapour absorption bands. The leaf variable that has the greatest contribution to the TOA radiance spectrum explains more than 50% of the variance in the whole spectrum. Chlorophyll content (Cab) has a dominant impact in the visible region but disappears throughout the red edge as the wavelengths become too large for chlorophyll absorption [94]. Dry matter content (Cm) is dominant in the NIR (750–1200 nm) and water content (Cw) in the SWIR (1400–500 nm). The results are very similar to the ones found by [93]. Regarding the canopy variables, the most important one was the leaf area index (LAI), especially in the visible range and SWIR, and less important in the NIR, also found in other studies [32,93]. A limitation in the conducted study is that soil brightness coefficient was not included in the GSA. As demonstrated in earlier studies [91], this variable also exerts some influence of a few percent, about equally spaced along the spectral range. The variables that shows hardly any sensitivity, e.g., N, can be safely kept to default values in order to simplify and speed up the GSA [91]. From a retrieval point of view, the GSA result determines which of TOA radiance input variables are the most relevant, and thus suitable for retrieval directly from TOA radiance. Given the dominance of Cab, Cd and Cw at the leaf scale and LAI at the canopy scale, in principle, these variables are retrievable from at-sensor TOA radiance data, as has been shown before [11].

### 5.3 TOC and TOA Retrieval Models

When it comes to the developed TOC and TOA retrieval models, the relevant bands as given by the GPR band sigmas (*σ_b_*) (Figure 6) are supposed to be in agreement with the obtained GSA results (Figure 5). The bands with lowest *σ_b_* are expected to fall within regions of high sensitivity towards the targeted variable.

- Regarding the Cab models, the most relevant bands (low *σ_b_*) for both TOC and TOA fall within the visible region which is justified by the high sensitivity of Cab. The *S_Ti_* rapidly declines when entering the red edge, which is also observed by the higher sigmas. Of interest hereby are the relatively high importance of the two SWIR bands, even though the GSA results show Cab has no influence there. This has to be interpreted by indirect co-varying relationships between LAI and Cab. After all, Cab absorption only occurs when leaves are available (which in turn reduce the role of soil background). The amount of leaves is controlled by LAI [53,87].
- Regarding the Cw models, the most relevant bands for both TOC and TOA are found in the 1610 and 2190 nm SWIR bands. These are regions where Cw plays an important role. Further, the *σ_b_* band ranking suggest that also the visible bands are of importance, which can be again attributed to co-varying relationships with other leaf properties such as Cab and the amount of leaves, i.e., LAI [53,87].
- Regarding the LAI models, relevant bands are found all throughout the spectra with lowest *σ_b_* in the red (665 nm), and especially in the two SWIR bands. This is again in agreement with the GSA results where LAI is dominant in the SWIR.

We subsequently applied the TOC and TOA models to S2 L1C and L2A subsets for mapping applications over the Barrax agricultural site. The obtained maps merely serve as proof of concept to demonstrate that retrievals can be directly obtained from L1C TOA radiance data, i.e., without the need for an atmospheric correction. While results are encouraging, it must be pointed out that the models are still premature to make them produce accurate estimates for each pixel. Validation against ground truth data and fine tuning of the models is still required, e.g., accounting for the diverse variability of non-vegetated surfaces present in a S2 image, yet that is considered as beyond the scope of this work. Here we merely present the streamlined processing framework for the development of vegetation properties retrieval models applicable to at-sensor TOA data made available to the community. In this respect, in view of applying the presented tools for mapping applications, there are some opportunities for improvements that deserve to be mentioned:

- Obtained maps from L1C and L2A data are surprisingly consistent given that no optimization steps were applied. Yet, it must be remarked, the images were acquired on a clear-sky summer day for a flat surface, making that the role of atmosphere is predominantly homogeneous and predictable. Obviously the retrieval from TOA data will be more challenging in a more rugged terrain and in atmospheric heterogeneous conditions, e.g., haze, clouds and shadowing effects. With the offered toolboxes (ALG, TOC2TOA, GSA, retrieval) these effects can be studied, and specific retrieval strategies developed.
- The TOC and TOA models were trained by simulated data using RTMs that deal with spectral variability of homogeneous vegetated surfaces. Although 20 soil spectral signatures were added to the training, that is definitely not enough to cover the natural variability of non-vegetated surfaces at S2 spatial resolution for complete images. For instance, the models are not trained for water bodies and man-made surfaces. Ideally, spectral variability of all kinds of non-vegetated surfaces should be added to the training dataset. Similarly, most likely the model performs poorly over heterogeneous vegetated surfaces such as forests.
- Another way how to further optimize the training LUT for operational mapping is by using sample distributions that reflect reality more, e.g., normal or log-normal distributions for key variables. A more refined LUT may be necessary to mitigate the drawback of the LAI saturation. It is expected that by refining the LUT, e.g., excluding unrealistic situations the LAI model will be greatly improved, e.g., that saturation only occurs at higher LAI (>6). This is also the strategy in the official S2 vegetation algorithms as found within the SNAP toolbox [95].
- There are some aspects of the obtained maps from L1C and L2A data that require clarification. For instance, the fact that L2A-retrieved Cab is more pronounced than the one from L1C might indicate that the atmosphere has still some impact on the Cab. Indeed, aerosol properties have some influence in the AOT (although according to the GSA results this influence is residual <5%). The same holds for LAI, since LAI is also sensitive to the visible part (not only in the SWIR). Regarding Cw, their similarities in the obtained L1C vs L2A maps can be explained from the GSA, since Cw is mostly impacting in the SWIR range, where outside the water absorption bands the atmosphere has little influence. In this respect, it can be understood that Cw achieves the same performance from L1C or from L2A data.
- As a final remark, the TOA reflectance to TOA radiance conversion as well the Sen2Cor TOA (L1C) to BOA (L2A) conversion is done with routines based on the libRadtran RTM. These differences may lead to discrepancies as compared to the here used MODTRAN routines. For instance, the S2 processing uses the Thuillier [96] solar irradiance, while MODTRAN uses the Kurucz [97]. The role of using different atmospheric RTMs in atmospheric correction and in TOA radiance biophysical variables retrieval is yet to be investigated.

Given these topics for improvements, it would be premature to apply the obtained models into an operational context, but that is also not the aim of this study, as here the tools have been created to facilitate the developments of hybrid (i.e., RTM-based) TOA retrieval algorithms. It is foreseen that in follow-up studies the processing chain will be applied for dedicated TOA-based mapping applications.

### 5.4 ARTMO Toolboxes

The retrieval of vegetation properties from at-sensor TOA radiance data was made possible thanks to the development of two new toolboxes integrated within the ARTMO framework: ALG and TOC2TOA. These toolboxes allow to streamline RTM simulations and do the coupling between canopy simulations and atmosphere RTMs. ALG generates look-up tables based on a suite of atmospheric RTMs (6SV, MODTRAN and Libratran) [19]. ARTMO already allowed to run vegetation RTMs in a forward and inverse direction at the leaf and canopy level. With the TOC2TOA toolbox the coupling with atmospheric LUTs has been made possible. Yet, here only the first version of the TOC2TOA toolbox has been presented, and new utilities and improvements are considered; e.g., (1) to take adjacency effects into account [17], (2) to couple surface with atmosphere for non-Lambertian surfaces [14], and (3) to add the possibility to couple atmospheric models with water RTMs.

Furthermore, ARTMO incorporates several RTM post-processing toolboxes such as the retrieval toolboxes and the Emulation and GSA toolboxes. By combining both toolboxes, multiple TOA sensitivity studies or retrieval strategies can be developed and analyzed, e.g., for all kinds of atmospheric scenarios. All the presented toolboxes are freely downloadable at http://artmotoolbox.com. They can facilitate the interested user to repeat the presented study or to conduct related at-sensor TOA radiance studies that involve the processing of RTM simulations, sensitivity, emulation or retrieval.

## 6 Conclusions

This study aimed to quantify the relative importance of key input variables in leaf, canopy and atmosphere radiative transfer models (RTM) by using Gaussian process regression as emulator. Such models can be used to derive top-of-atmosphere radiance data that can be further used to estimate biophysical variables. To do so, the leaf RTM PROSPECT-4 was coupled with the canopy RTM SAIL and the atmosphere RTM MODTRAN. Because MODTRAN is computationally expensive, a bypass was sought by making use of emulation. Emulators are statistical constructs that enable to approximate the outputs of the original RTMs, but this is at low computation cost so that large LUTs can be produced almost instantly. The emulator subsequently allowed to calculate a global sensitivity analysis (GSA) and to identify the driving variables. The GSA total sensitivity index quantified that vegetation variables had a more dominant impact than atmosphere variables on TOA radiance for atmospheric windows. This finding provides support to develop retrieval strategies of biophysical variables such as leaf chlorophyll content (Cab), leaf water content (Cw) and leaf area index (LAI) directly from TOA radiance data, e.g., given Sentinel-2 band settings.

Accordingly, the coupled leaf-canopy-atmosphere RTMs served to train hybrid retrieval models by using the machine learning algorithm Gaussian processes regression for the processing of Sentinel-2 TOA radiance data (L1C) and bottom-of-atmosphere reflectance data (L2A) given a cloud-free sky. Retrievals of Cab, Cw and LAI were consistent, although optimization is still required for operational processing. The maps demonstrate the possibility to retrieve biophysical variables directly from at-sensor TOA radiance data by means of developing machine learning models, thus without the need of an atmospheric correction step, and this in a streamlined and largely automated environment.

Summarizing, to the benefit of the community, the here developed toolboxes enable the coupling of leaf-canopy-atmosphere RTMs for any sensor band settings, so they can be used for the generation of TOA LUTs for multiple Earth observation applications, e.g., the retrieval of surface and atmospheric variables.

## Figures and Tables

**Table 1:** MSI/Sentinel-2 sensor characteristics as per [41].

Technical Characteristics	Value
Imaging principle	Pushbroom-grating
Spectral range [nm]	400–2200 nm
Geolocation accuracy	<12.5 m
SNR @L_*ref*_	50–175
Radiometric accuracy	3% abs, 1% rel
A/D conversion	12 bits
Coverage	Land and coastal areas

**Table 2:** Range of vegetation input variables for the PROSAIL LUTs according to Latin Hypercube sampling. SAIL fixed variables: hot spot: 0.01; solar zenith angle: 30°; observer zenith angle: 0°.

Model Variables		Units	Minimum	Maximum
**Leaf variables (PROSPECT-4)**				
N	Leaf structure index	unitless	1.3	2.5
Cw	Leaf water content	[g/cm^2^] or [cm]	0.002	0.05
Cab	Leaf chlorophyll content	[μg/cm^2^]	1	70
Cm	Leaf dry matter content	[g/cm^2^]	0.002	0.05
**Canopy variables (SAIL)**				
LAI	Leaf area index	[m^2^/m^2^]	0.1	7
LAD	Leaf angle distribution	[°]	0	90

**Table 3:** Range of MODTRAN input variables for the *analysis* dataset according to Latin Hypercube sampling. MODTRAN fixed geometric variables: solar zenith angle: 30°; viewing zenith angle: 0°. Remaining MODTRAN parameters were set to their default values.

	Model Variables	Units	Minimum	Maximum
O3C	O_3_ column concentration	[amt-cm]	0.25	0.35
CWV	Columnar Water Vapour	g·cm^–2^	0.4	4.5
AOT_550_	Aerosol Optical Thickness at 550 nm	unitless	0.05	0.5
G	Asymmetry parameter	unitless	0.6	1
*α*	Ångström exponent	unitless	0.05	2
SSA	Single Scattering Albedo	unitless	0.85	1

**Table 4:** Range of MODTRAN input variables for the *reference_toa* subset according to Latin Hypercube sampling. MODTRAN fixed geometric variables: solar zenith angle: 30°; viewing zenith angle: 0°. Remaining MODTRAN parameters were set to their default values.

	Model Variables	Units	Minimum	Maximum
O3C	O_3_ column concentration	[amt-cm]	0.25	0.35
CWV	Columnar Water Vapour	g · cm^–2^	0.4	4.5
AOT_550_	Aerosol Optical Thickness at 550 nm	unitless	0.05	0.5
	Aerosol type	9 types (see text above)		

**Table 5:** Retrieval performance results against 5’000 LUT *reference* datasets for biophysical variables retrieval from surface reflectance (TOC), TOA radiance (TOA) and surface reflectance with noise levels after atmospheric correction (ATM). For the TOC and TOA *retrieval* datasets 2% Gaussian noise was added, while for the TOC-ATM *retrieval* datasets noises are added according to [50].

Retrieval	TOC	TOA	TOC-ATM
**R** ^2^ **:**			
- Cab	0.972	0.948	0.907
- Cw	0.942	0.908	0.813
- LAI	0.684	0.623	0.520
**RMSE:**			
- Cab	3.312	4.586	6.077
- Cw	0.003	0.004	0.006
- LAI	1.120	1.223	1.381

## References

[1] Malenovský Z, Rott H, Cihlar J, Schaepman ME, García-Santos G, Fernandes R, Berger M (2012). Sentinels for science: Potential of Sentinel-1, -2, and -3 missions for scientific observations of ocean, cryosphere, and land. Remote Sens Environ.

[2] Verrelst J, Rivera J, Van Der Tol C, Magnani F, Mohammed G, Moreno J (2015). Global sensitivity analysis of the SCOPE model: What drives simulated canopy-leaving sun-induced fluorescence?. Remote Sens Environ.

[3] Drusch M, Del Bello U, Carlier S, Colin O, Fernandez V, Gascon F, Hoersch B, Isola C, Laberinti P, Martimort P (2012). Sentinel-2: ESA’s Optical High-Resolution Mission for GMES Operational Services. Remote Sens Environ.

[4] North P, Brockmann C, Fischer J, Gomez-Chova L, Grey W, Heckel A, Moreno J, Preusker R, Regner P MERIS/AATSR synergy algorithms for cloud screening, aerosol retrieval and atmospheric correction.

[5] Main-Knorn M, Pflug B, Louis J, Debaecker V, Mueller-Wilm U, Gascon F Sen2Cor for sentinel-2.

[6] Richter R, Schläpfer D (2002). Geo-atmospheric processing of airborne imaging spectrometry data. Part 2: Atmospheric/topographic correction. Int J Remote Sens.

[7] Guanter L, González-Sanpedro MDC, Moreno J (2007). A method for the atmospheric correction of ENVISAT/MERIS data over land targets. Int J Remote Sens.

[8] Holben B, Eck T, Slutsker I, Tanré D, Buis J, Setzer A, Vermote E, Reagan J, Kaufman Y, Nakajima T (1998). AERONET—A federated instrument network and data archive for aerosol characterization. Remote Sens Environ.

[9] Dee DP, Uppala SM, Simmons AJ, Berrisford P, Poli P, Kobayashi S, Andrae U, Balmaseda MA, Balsamo G, Bauer P (2011). The ERA-Interim reanalysis: Configuration and performance of the data assimilation system. Q J R Meteorol Soc.

[10] Kokhanovsky A, Breon FM, Cacciari A, Carboni E, Diner D, Di Nicolantonio W, Grainger R, Grey W, Höller R, Lee KH (2007). Aerosol remote sensing over land: A comparison of satellite retrievals using different algorithms and instruments. Atmos Res.

[11] Laurent V, Verhoef W, Clevers J, Schaepman M (2011). Inversion of a coupled canopy-atmosphere model using multi-angular top-of-atmosphere radiance data: A forest case study. Remote Sens Environ.

[12] Laurent V, Verhoef W, Clevers J, Schaepman M (2011). Estimating forest variables from top-of-atmosphere radiance satellite measurements using coupled radiative transfer models. Remote Sens Environ.

[13] Laurent V, Verhoef W, Damm A, Schaepman M, Clevers J (2013). A Bayesian object-based approach for estimating vegetation biophysical and biochemical variables from APEX at-sensor radiance data. Remote Sens Environ.

[14] Mousivand A, Menenti M, Gorte B, Verhoef W (2015). Multi-temporal, multi-sensor retrieval of terrestrial vegetation properties from spectral-directional radiometric data. Remote Sens Environ.

[15] Shi H, Xiao Z, Liang S, Ma H (2017). A method for consistent estimation of multiple land surface parameters from MODIS top-of-atmosphere time series data. IEEE Trans Geosci Remote Sens.

[16] Fourty T, Baret F (1997). Vegetation water and dry matter contents estimated from top-of-the-atmosphere reflectance data: A simulation study. Remote Sens Environ.

[17] Verhoef W, Bach H (2003). Simulation of hyperspectral and directional radiance images using coupled biophysical and atmospheric radiative transfer models. Remote Sens Environ.

[18] Verhoef W, Bach H (2007). Coupled soil-leaf-canopy and atmosphere radiative transfer modeling to simulate hyperspectral multi-angular surface reflectance and TOA radiance data. Remote Sens Environ.

[19] Vicent J, Sabater N, Verrelst J, Alonso L, Moreno J (2017). Assessment of Approximations in Aerosol Optical Properties and Vertical Distribution into FLEX Atmospherically-Corrected Surface Reflectance and Retrieved Sun-Induced Fluorescence. Remote Sens.

[20] Feret JB, François C, Asner GP, Gitelson AA, Martin RE, Bidel LPR, Ustin SL, le Maire G, Jacquemoud S (2008). PROSPECT-4 and 5: Advances in the leaf optical properties model separating photosynthetic pigments. Remote Sens Environ.

[21] Verhoef W (1984). Light scattering by leaf layers with application to canopy reflectance modeling: The SAIL model. Remote Sens Environ.

[22] Jacquemoud S, Verhoef W, Baret F, Bacour C, Zarco-Tejada P, Asner G, François C, Ustin S (2009). PROSPECT + SAIL models: A review of use for vegetation characterization. Remote Sens Environ.

[23] Berger K, Atzberger C, Danner M, D’Urso G, Mauser W, Vuolo F, Hank T (2018). Evaluation of the PROSAIL model capabilities for future hyperspectral model environments: A review study. Remote Sens.

[24] Berk A, Bernstein L, Anderson G, Acharya P, Robertson D, Chetwynd J, Adler-Golden S (1998). MODTRAN cloud and multiple scattering upgrades with application to AVIRIS. Remote Sens Environ.

[25] Berk A, Conforti P, Kennett R, Perkins T, Hawes F, van den Bosch J MODTRAN6: A major upgrade of the MODTRAN radiative transfer code.

[26] Schaepman M, Ustin S, Plaza A, Painter T, Verrelst J, Liang S (2009). Earth system science related imaging spectroscopy-An assessment. Remote Sens Environ.

[27] Homolová L, Malenovský Z, Clevers J, García-Santos G, Schaepman M (2013). Review of optical-based remote sensing for plant trait mapping. Ecol Complex.

[28] Saltelli A, Ratto M, Andres T, Campolongo F, Cariboni J, Gatelli D, Saisana M, Tarantola S (2008). Global Sensitivity Analysis: The Primer;.

[29] Saltelli A (2002). Making best use of model evaluations to compute sensitivity indices. Comput Phys Commun.

[30] Kotchenova SY, Vermote EF, Levy R, Lyapustin A (2008). Radiative transfer codes for atmospheric correction and aerosol retrieval: Intercomparison study. Appl Optics.

[31] Rivera JP, Verrelst J, Gómez-Dans J, Muñoz Marí J, Moreno J, Camps-Valls G (2015). An Emulator Toolbox to Approximate Radiative Transfer Models with Statistical Learning. Remote Sens.

[32] Verrelst J, van der Tol C, Magnani F, Sabater N, Rivera J, Mohammed G, Moreno J (2016). Evaluating the predictive power of sun-induced chlorophyll fluorescence to estimate net photosynthesis of vegetation canopies: A SCOPE modeling study. Remote Sens Environ.

[33] Verrelst J, Rivera Caicedo J, Muñoz Marí J, Camps-Valls G, Moreno J (2017). SCOPE-based emulators for fast generation of synthetic canopy reflectance and sun-induced fluorescence Spectra. Remote Sens.

[34] Petropoulos G, Wooster M, Carlson T, Kennedy M, Scholze M (2009). A global Bayesian sensitivity analysis of the 1D SimSphere soil vegetation atmospheric transfer (SVAT) model using Gaussian model emulation. Ecol Model.

[35] Rohmer J, Foerster E (2011). Global sensitivity analysis of large-scale numerical landslide models based on Gaussian-Process meta-modeling. Comput Geosci.

[36] Bounceur N, Crucifix M, Wilkinson RD (2014). Global sensitivity analysis of the climate–vegetation system to astronomical forcing: An emulator-based approach. Earth Syst Dyn Discuss.

[37] Ryan E, Wild O, Voulgarakis A, Lee L (2018). Fast sensitivity analysis methods for computationally expensive models with multi-dimensional output. Geosci Model Dev.

[38] Verrelst J, Camps Valls G, Muñoz Marí J, Rivera J, Veroustraete F, Clevers J, Moreno J (2015). Optical remote sensing and the retrieval of terrestrial vegetation bio-geophysical properties—A review. ISPRS J Photogramm Remote Sens.

[39] Verrelst J, Malenovský Z, van der Tol C, Camps-Valls G, Gastellu-Etchegorry JP, Lewis P, North P, Moreno J (2018). Quantifying Vegetation Biophysical Variables from Imaging Spectroscopy Data: A Review on Retrieval Methods. Surv Geophys.

[40] Pflug B, Main-Knorn M, Bieniarz J, Debaecker V, Louis J Early validation of sentinel-2 L2A processor and products.

[41] ESA Sentinel-2 MSI Technical Guide.

[42] ESA (2018). Sentinel-2 Spectral Response Functions (S2-SRF), v30, Ref: COPE-GSEG-EOPG-TN-15-0007; Technical Report.

[43] Richter R, Wang X, Bachmann M, Schläpfer D (2011). Correction of cirrus effects in Sentinel-2 type of imagery. Int J Remote Sens.

[44] Louis J, Charantonis A, Berthelot B Cloud Detection for Sentinel-2.

[45] Kaufman Y, Sendra C (1988). Algorithm for automatic atmospheric corrections to visible and near-IR satellite imagery. Int J Remote Sens.

[46] Schläpfer D, Borel C, Keller J, Itten K (1998). Atmospheric Precorrected Differential Absorption Technique to Retrieve Columnar Water Vapor. Remote Sens Environ.

[47] Emde C, Buras-Schnell R, Kylling A, Mayer B, Gasteiger J, Hamann U, Kylling J, Richter B, Pause C, Dowling T (2016). The libRadtran software package for radiative transfer calculations (version 2.0.1). Geosci Model Dev.

[48] Louis J, Debaecker V, Pflug B, Main-Knorn M, Bieniarz J, Mueller-Wilm U, Cadau E, Gascon F Sentinel-2 SEN2COR: L2A processor for users.

[49] Martins VS, Barbosa CCF, de Carvalho LAS, Jorge DSF, Lobo FdL, Novo EMLDM (2017). Assessment of Atmospheric Correction Methods for Sentinel-2 MSI Images Applied to Amazon Floodplain Lakes. Remote Sens.

[50] Li Y, Chen J, Ma Q, Zhang H, Liu J (2018). Evaluation of Sentinel-2A Surface Reflectance Derived Using Sen2Cor in North America. IEEE J Sel Top Appl Earth Obs Remote Sens.

[51] Vuolo F, Zółtak M, Pipitone C, Zappa L, Wenng H, Immitzer M, Weiss M, Baret F, Atzberger C (2016). Data Service Platform for Sentinel-2 Surface Reflectance and Value-Added Products: System Use and Examples. Remote Sens.

[52] Djamai N, Fernandes R, Weiss M, McNairn H, Goïta K (2019). Validation of the Sentinel Simplified Level 2 Product Prototype Processor (SL2P) for mapping cropland biophysical variables using Sentinel-2/MSI and Landsat-8/OLI data. Remote Sens Environ.

[53] Verrelst J, Alonso L, Camps-Valls G, Delegido J, Moreno J (2012). Retrieval of vegetation biophysical parameters using Gaussian process techniques. IEEE Trans Geosci Remote Sens.

[54] Guanter L, Richter R, Kaufmann H (2009). On the application of the MODTRAN4 atmospheric radiative transfer code to optical remote sensing. Int J Remote Sens.

[55] Verrelst J, Rivera JP, Moreno J (2015). ARTMO’s global sensitivity analysis (GSA) toolbox to quantify driving variables of leaf and canopy radiative transfer models. EARSeL eProceedings Speical.

[56] Caicedo J, Verrelst J, Munoz-Mari J, Moreno J, Camps-Valls G (2014). Toward a semiautomatic machine learning retrieval of biophysical parameters. IEEE J Sel Top Appl Earth Obs Remote Sens.

[57] Berk A, Anderson G, Acharya P, Bernstein L, Muratov L, Lee J, Fox M, Adler-Golden S, Chetwynd J, Hoke M MODTRAN™5: 2006 update.

[58] Cooley T, Anderson G, Felde G, Hoke M, Ratkowski A, Chetwynd J, Gardner J, Adler-Golden S, Matthew M, Berk A (2002). FLAASH, a MODTRAN4-based atmospheric correction algorithm, its applications and validation.

[59] Stamnes K, Tsay SC, Wiscombe W, Jayaweera K (1988). Numerically stable algorithm for discrete-ordinate-method radiative transfer in multiple scattering and emitting layered media. Appl Opt.

[60] Goody R, West R, Chen L, Crisp D (1989). The correlated-k method for radiation calculations in nonhomogeneous atmospheres. J Quant Spectrosc Radiat Transf.

[61] McKay M, Beckman R, Conover W (1979). Comparison of three methods for selecting values of input variables in the analysis of output from a computer code. Technometrics.

[62] Hess M, Koepke P, Schult I (1998). Optical Properties of Aerosols and Clouds: The Software Package OPAC. Bull Am Meteorol Soc.

[63] Dubovik O, Holben B, Eck T, Smirnov A, Kaufman Y, King M, Tanré D, Slutsker I (2002). Variability of absorption and optical properties of key aerosol types observed in worldwide locations. J Atmos Sci.

[64] Spectral Sciences I Official MODTRAN6 Webpage.

[65] Yang J (2011). Convergence and uncertainty analyses in Monte-Carlo based sensitivity analysis. Environ Model Softw.

[66] Sobol’ IM (1990). On sensitivity estimation for nonlinear mathematical models. Mat Modelirovanie.

[67] Saltelli A, Annoni P, Azzini I, Campolongo F, Ratto M, Tarantola S (2010). Variance based sensitivity analysis of model output. Design and estimator for the total sensitivity index. Comput Phys Commun.

[68] Song K, Lu D, Li L, Li S, Wang Z, Du J (2012). Remote sensing of chlorophyll-a concentration for drinking water source using genetic algorithms (GA)-partial least square (PLS) modeling. Ecol Inform.

[69] Nossent J, Elsen P, Bauwens W (2011). Sobol’sensitivity analysis of a complex environmental model. Environ Model Softw.

[70] Saltelli A, Annoni P (2010). How to avoid a perfunctory sensitivity analysis. Environ Model Softw.

[71] O’Hagan A (2006). Bayesian analysis of computer code outputs: A tutorial. Reliab Eng Syst Saf.

[72] Hughes G (1968). On The Mean Accuracy Of Statistical Pattern Recognizers. IEEE Trans Inf Theory.

[73] Wold S, Esbensen K, Geladi P (1987). Principal component analysis. Chemom Intell Lab Syst.

[74] Liu X, Smith W, Zhou D, Larar A (2006). Principal component-based radiative transfer model for hyperspectral sensors: Theoretical concept. Appl Opt.

[75] Matricardi M (2010). A principal component based version of the RTTOV fast radiative transfer model. Q J R Meteorol Soc.

[76] Rivera-Caicedo JP, Verrelst J, Muñoz-Marí J, Camps-Valls G, Moreno J (2017). Hyperspectral dimensionality reduction for biophysical variable statistical retrieval. ISPRS J Photogramm Remote Sens.

[77] Gómez-Dans JL, Lewis PE, Disney M (2016). Efficient Emulation of Radiative Transfer Codes Using Gaussian Processes and Application to Land Surface Parameter Inferences. Remote Sens.

[78] Rasmussen CE, Williams CKI (2006). Gaussian Processes for Machine Learning.

[79] Bastos LS, O’Hagan A (2009). Diagnostics for Gaussian Process Emulators. Technometrics.

[80] Conti S, Gosling J, Oakley J, O’Hagan A (2009). Gaussian process emulation of dynamic computer codes. Biometrika.

[81] Liu F, West M (2009). A dynamic modelling strategy for bayesian computer model emulation. Bayesian Anal.

[82] Shawe-Taylor J, Cristianini N (2004). Kernel Methods for Pattern Analysis.

[83] Camps-Valls G, Bruzzone L (2009). Kernel Methods for Remote Sensing Data Analysis.

[84] Rojo-Álvarez J, Martínez-Ramón M, Muñoz Marí J, Camps-Valls G (2017). Digital Signal Processing with Kernel Methods.

[85] Camps-Valls G, Verrelst J, Munoz-Mari J, Laparra V, Mateo-Jimenez F, Gomez-Dans J (2016). A Survey on Gaussian Processes for Earth-Observation Data Analysis: A Comprehensive Investigation. IEEE Geosci Remote Sens Mag.

[86] Verrelst J, Rivera J, Veroustraete F, Muñoz Marí J, Clevers J, Camps-Valls G, Moreno J (2015). Experimental Sentinel-2 LAI estimation using parametric, non-parametric and physical retrieval methods—A comparison. ISPRS J Photogramm Remote Sens.

[87] Verrelst J, Muñoz J, Alonso L, Delegido J, Rivera J, Camps-Valls G, Moreno J (2012). Machine learning regression algorithms for biophysical parameter retrieval: Opportunities for Sentinel-2 and -3. Remote Sens Environ.

[88] Verrelst J, Rivera J, Moreno J, Camps-Valls G (2013). Gaussian processes uncertainty estimates in experimental Sentinel-2 LAI and leaf chlorophyll content retrieval. ISPRS J Photogramm Remote Sens.

[89] Baret F, Hagolle O, Geiger B, Bicheron P, Miras B, Huc M, Berthelot B, Niño F, Weiss M, Samain O (2007). LAI, fAPAR and fCover CYCLOPES global products derived from VEGETATION. Part 1: Principles of the algorithm. Remote Sens Environ.

[90] Vicent J, Verrelst J, Rivera-Caicedo JP, Sabater N, Muñoz Marí J, Camps-Valls G, Moreno J (2018). Emulation as an Alternative to Interpolation in Sampling Radiative Transfer Codes. IEEE J Sel Top Appl Earth Obs Remote Sens.

[91] Verrelst J, Sabater N, Rivera JP, Muñoz Marí J, Vicent J, Camps-Valls G, Moreno J (2016). Emulation of Leaf, Canopy and Atmosphere Radiative Transfer Models for Fast Global Sensitivity Analysis. Remote Sens.

[92] Myneni R, Asrar G (1994). Atmospheric effects and spectral vegetation indices. Remote Sens Environ.

[93] Mousivand A, Menenti M, Gorte B, Verhoef W (2014). Global sensitivity analysis of the spectral radiance of a Soil-vegetation system. Remote Sens Environ.

[94] Curran PJ, Dungan JL, Gholz HL (1990). Exploring the relationship between reflectance red edge and chlorophyll content in slash pine. Tree Physiol.

[95] Weiss M, Baret F (2016). ESA Contract nr 4000110612/14/I-BG.

[96] Thuillier G, Hersé M, Foujols T, Peetermans W, Gillotay D, Simon P, Mandel H (2003). The solar spectral irradiance from 200 to 2400 nm as measured by the SOLSPEC spectrometer from the ATLAS and EURECA missions. Sol Phys.

[97] Chance K, Kurucz RL (2010). An improved high-resolution solar reference spectrum for earth’s atmosphere measurements in the ultraviolet, visible, and near infrared. J Quant Spectrosc Radiat Transf.

